# Dengue infection triggered immune mediated necrotizing myopathy in children: a case report and literature review

**DOI:** 10.1186/s12969-022-00699-2

**Published:** 2022-06-07

**Authors:** Aphirak Mekmangkonthong, Jakkrit Amornvit, Numphung Numkarunarunrote, Montida Veeravigrom, Parichat Khaosut

**Affiliations:** 1 Division of Paediatric Neurology, Department of Paediatrics, Faculty of Medicine, Chulalongkorn University, Bangkok, Thailand; 2 Division of Paediatric Neurology, Department of Paediatrics, King Chulalongkorn Memorial Hospital/The Thai Red Cross Society, Bangkok, Thailand; 3 Division of Neurology, Department of Medicine, Faculty of Medicine, Chulalongkorn University, Bangkok, Thailand; 4 Division of Neurology, Department of Medicine, Faculty of Medicine, King Chulalongkorn Memorial Hospital/The Thai Red Cross Society, Bangkok, Thailand; 5 Department of Radiology, Faculty of Medicine, Chulalongkorn University, Bangkok, Thailand; 6 Section of Child Neurology, Department of Paediatrics, Comer Children’s Hospital, The University of Chicago, Chicago, IL USA; 7 Division of Paediatric Allergy, Immunology and Rheumatology, Department of Paediatrics, Faculty of Medicine, Chulalongkorn University, Bangkok, Thailand; 8 Center of Excellence for Allergy and Clinical Immunology, Division of Paediatric Allergy, Immunology and Rheumatology, Department of Paediatrics, Faculty of Medicine, Chulalongkorn University, King Chulalongkorn Memorial Hospital/The Thai Red Cross Society, Bangkok, Thailand; 9 Division of Allergy and Immunology, Department of Pediatrics, Faculty of Medicine, Chulalongkorn University, King Chulalongkorn Memorial Hospital, 1873 Rama IV Road, Pathum Wan District, Bangkok, 10330 Thailand

**Keywords:** Immune-mediated necrotizing myopathy, Anti-3-hydroxy-3-methylglutaryl-CoA reductase (anti-HMGCR), Inflammatory myopathy, Paediatric, dengue

## Abstract

**Background:**

Immune-mediated necrotizing myopathy (IMNM) is a subgroup of idiopathic inflammatory myopathies manifesting with progressive weakness, elevated serum creatine kinase (CK) levels, and necrotizing myopathic features on muscle biopsy. There is a paucity of data on the clinical presentation of IMNM in children. We report a paediatric patient who developed anti-3-hydroxy-3-methylglutaryl-CoA reductase (anti-HMGCR)-positive necrotizing myopathy after recent dengue infection.

**Case presentation:**

A previously healthy 9-year-old boy presented with acute proximal muscle weakness after recovery from dengue infection. Five days after the fever subsided, he could not stand from a squatting position. He denied having skin rash, arthritis, or other systemic features. He had marked elevation of CK level of 30,833 mg/dL and was put on steroid therapy. The patient initially responded to oral prednisolone, however the weakness persisted and muscle enzymes increased as steroids were decreased. He was then referred to our hospital for further assessment. Subsequent investigation revealed anti-HMGCR positivity along with specific histopathological findings consistent with IMNM. The patient was treated with six cycles of intravenous immunoglobulin (IVIG) monthly, then followed by a gradual taper of prednisolone and oral methotrexate weekly with complete recovery in motor power.

**Conclusions:**

Our report presents a child with clinical manifestations of IMNM which can be categorized as acute onset of muscle weakness following dengue infection. Two key points supporting a diagnosis in this case are clinical response after immunosuppressive therapy and absence of rashes found in juvenile dermatomyositis.

## Rheumatology key message

• IMNM in children can be categorized as “acute onset” of weakness following a viral infection.

• Testing for relevant myositis autoantibodies may help improve diagnostic accuracy and allow for timely treatment with highly effective immunotherapies.

Immune-mediated necrotizing myopathy (IMNM) is a recently recognized category of idiopathic inflammatory myopathies manifesting as symmetric proximal muscle weakness and high serum creatine kinase (CK) levels, along with specific histopathological findings [1]. There is a current paucity of data on the clinical phenotype of IMNM in the paediatric population. In this report, we described a boy with anti-3-hydroxy-3-methylglutaryl-CoA reductase (anti-HMGCR)-positive necrotizing myopathy who presented with acute onset of weakness after recent dengue infection.

This case involves a 9-year-old Thai boy who was previously healthy before developing high grade fever with myalgia for 4 consecutive days. During the resolution of the fever, erythematous itchy rashes on lower limbs resembling convalescent rashes in dengue infection were observed. He was diagnosed with dengue fever 11 weeks before referral to our hospital. He had positive serology with dengue IgM and IgG. A test for SAR-COV2 infection was not completed at that time because all of his symptoms developed in March 2019 prior to the pandemic. Five days after the fever subsided, he had proximal muscle weakness in the bilateral lower extremities so that he could no longer stand from a squatting position. He needed assistance to stand first before being able to walk. He denied any weakness in his upper extremities. He denied having any muscle pain or difficulty swallowing. The family also denied any use of herbal medicines or other toxic exposures. His family did not have any significant history of conditions such as neuromuscular and autoimmune disease. His physical examination revealed normal mental status and cranial nerve II-XII function. Motor power measured by the medical research council classification of bilateral proximal and distal upper extremities was grade 4/5 and 5/5, respectively. The proximal and distal muscle strength of his lower extremities were grade 3/5 and 4/5, respectively. Deep tendon reflexes were 2+ all. Serum creatine kinase level (CK) was significantly elevated at 30,833 mg/dL (normal 55–324 mg/dL).

Intravenous fluid hydration and prednisolone 2 mg/kg/day were started during admission. Prednisolone was tapered off within 3 weeks. His muscle strength was improved to grade 4/5 at bilateral upper and lower extremities. His CK level was decreased to 7787 mg/dL at the time of weaning prednisolone. Seven days after stopping prednisolone, without any changes in motor power, his CK level increased back up to 16,895 mg/dL. The medication was reinitiated and he was referred to our tertiary care hospital for further assessment.

Our physical examination revealed no skin rash, joint swelling, or other systemic features. A neurological examination did not demonstrate muscle wasting, muscle hypertrophy or pseudohypertrophy. He had neck flexors power grade 3/5, Gowers sign positive, and Trendelenburg sign positive. His motor strength is shown in Table 1.

**Table 1:** Muscle power of patient at the time of evaluation

Upper extremities	Right	Left
Shoulder flexors	3/5	3/5
Shoulder extensors	3/5	3/5
Shoulder abduction	3/5	3/5
Elbow flexion, extension	4/5, 4/5	4/5, 4/5
Wrist flexion, extension	5/5, 5/5	5/5, 5/5
Abductor pollicis brevis	4/5	4/5
Abductor digiti minimi	4/5	4/5
Lower extremities	Right	Left
Hip flexors	2/5	2/5
Hip extensors	2/5	2/5
Hip Abduction, adduction	4/5, 4/5	4/5, 4/5
Knee flexors, extensors	4/5, 4/5	4/5, 4/5
Ankle dorsi/ plantar flexion	4/5, 4/5	4/5, 4/5
Extensor hollicis longus	4/5	4/5

His CK level was decreased to 5051 mg/dL after reintroduction of prednisolone for one month. He had an elevated C-reactive protein of 1.2 mg/dL (normal 0–0.5 mg/dL), erythrocyte sedimentation rate of 30 mm/hr. (normal 0–15 mm/hr) and lactate dehydrogenase of 977 U/L (normal 192–232 U/L). His aspartate aminotransferase was 175 U/L (normal 18–36 U/L) and alanine transferase was 286 U/L (normal 10–35 U/L). A test for antibodies to HMGCR was positive but other myositis specific and/or associated antibodies* were all negative including the anti-signal recognition particle antibody (anti SRP). His anti-nuclear antibody, thyroid function, thyroid antibody, complete blood count, electrolyte, blood urea nitrogen, creatinine, and tumor markers were also all normal.

**Fig. 1:**
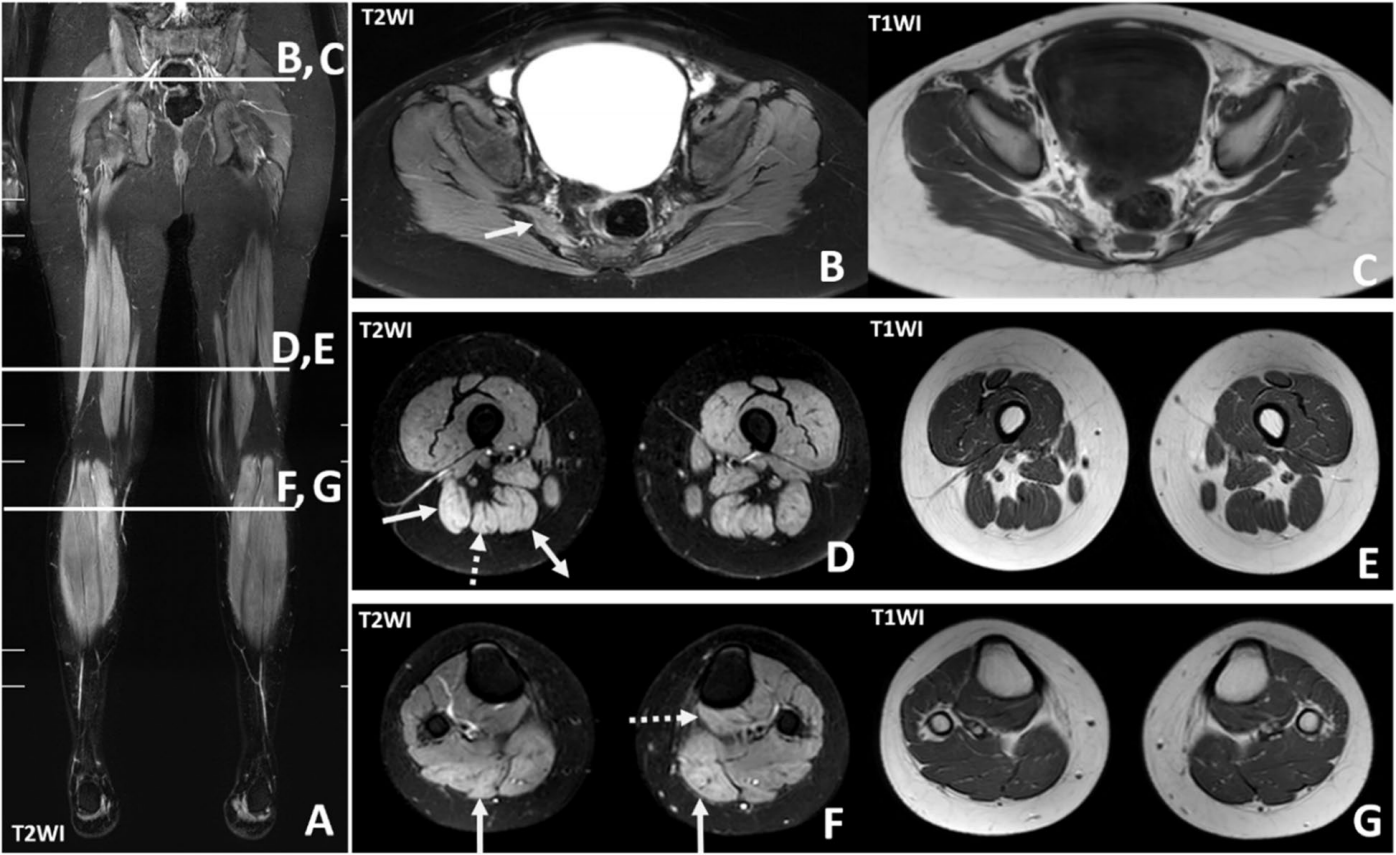
Magnetic resonance imaging (MRI) of lower extremities. **A** Coronal T2-weighted image (T2WI) **B**, **D**, **F**: Axial T2WI and **C**, **E**, **G**: Axial T1WI. The axial and coronal images show increased signal intensity on T2WI of right piriformis muscle (solid arrow in **B**), right long head of biceps femoris muscle (solid arrow in **D**), right semitendinosus muscle (dash arrow in **D**), right semimembranosus muscle (double-head arrow in **D**), bilateral gastrocnemius muscles (solid arrow in **F**) and left popliteus muscle (dash arrow in **F**)

**Fig. 2:**
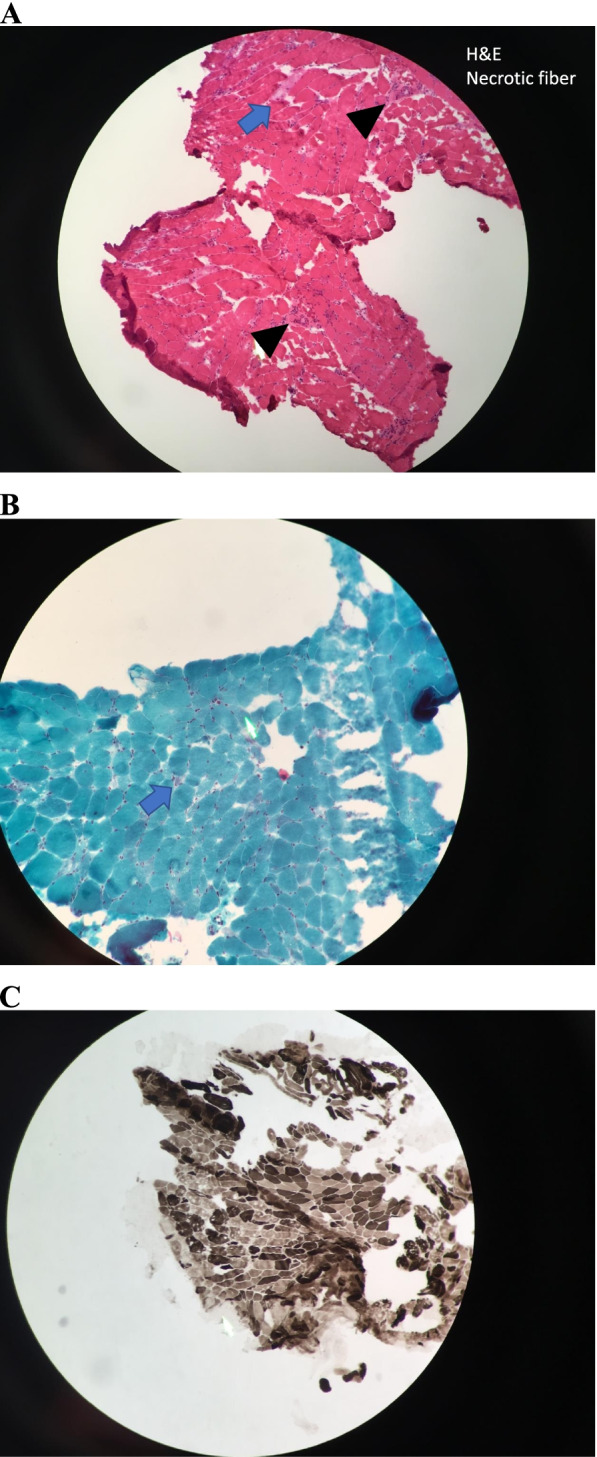
Muscle biopsy revealed necrotic fiber (blue arrow) and perivascular lymphocytosis (arrowhead) on hematoxylin and eosin (**H**&**E**) stain (**A**). Necrotic tissue on gomori trichrome stain (blue arrow) (**B**). Type 1 and type 2 muscle atrophy on ATP stain (**C**)

Nerve conduction study was normal. His electromyography revealed an inflammatory myopathic pattern. Magnetic resonance imaging (MRI) of lower extremities showed asymmetrical heterogeneous and increased signal intensity (SI) on T2-weighted image (T2WI) of the right piriformis, right long head of biceps femoris, right semitendinosus, right semimembranosus, bilateral gastrocnemius and left popliteus muscles, which could be myositis or myopathy. There was no atrophic change in the affected muscles or evidence of fluid collection along the adjacent superior fascia or deep fascia (Fig. 1). Muscle biopsy of the left gastrocnemius was performed and revealed necrotizing myopathy (Fig. 2).

Other investigations including electrocardiogram, echocardiogram, pulmonary function tests, chest X-ray and ultrasonography of abdomen were unremarkable. Methyl prednisolone of 1 g per day was started for 3 days followed by gradually reduced oral prednisolone. Two doses of 1 g of intravenous immunoglobulin (IVIG) per kg body weight on two consecutive days was administered monthly for 6 cycles. Although his motor power returned to grade 5/5 on all extremities, his creatine kinase remained elevated at the level of 1353 mg/dL. As his clinical progression showed an inadequate response to IVIG, oral methotrexate (MTX) at 15 mg/m^2^BSA weekly was later initiated at maintenance therapy alongside an oral steroid. At last follow-up, he remained well with the ability to do normal daily activities without any problem. MTX was optimized to higher dose of 25 mg subcutaneous injection weekly during the past few months to achieve optimal disease control. The patient is currently on close medical observation in our clinic.

## Discussion

Dengue is one of the most common mosquito-borne viral infections in Thailand and other tropical and subtropical countries. The average annual incidence is 115 cases per 100,000 population (72,000 cases/year, 100 death/year) in Thailand [2]. High-grade fever with myalgia is the common presentation of dengue infection. Viral myositis (otherwise known as “myalgia cruris”, “acute benign calf myositis”, “benign acute childhood myositis”) is an atypical neuromuscular manifestation in paediatric dengue infection and usually a self-limited condition [3, 4].

Our patient developed acute weakness at five days after recovery from dengue infection. The peculiarity of this case is the onset of weakness with an absence of myalgia. Acute pure motor weakness, one of the neurological manifestations, was reported during the second to fifth day of fever [5]. The patient initially responded to oral steroids, however the weakness persisted and the CK level increased as steroids were decreased. The patient did not have any signs of dermatomyositis. He only had transient rashes, which were compatible with convalescent rashes in dengue infection. His electromyography revealed irritable myopathic changes. The MRI showed increased SI on T2WI involving right posterior compartment of the thigh, bilateral gastrocnemius, left popliteus muscles and muscle pathology consistent with IMNM. We excluded the diagnosis of muscular dystrophy in this case because of clinical response following immunosuppressive therapy and a specific pattern of muscle biopsy with major histocompatibility complex (MHC) class I widespread overexpression in sarcolemmal.

IMNM is a very rare and distinctive type of inflammatory myopathy. The muscle pathology in this group shows necrotizing muscle fibers with minimal inflammatory cell infiltrates. The subtype of IMNM is determined by autoantibody testing into one of three subtypes: anti-SRP, anti-HMGCR or autoantibody negative [6]. Our patient had anti-HMGCR positivity which responded to immunosuppressive medication which allowed the patient to return to normal daily baseline activity. However, his CK was still persistently high after finishing 6 cycles of IVIG. The clinical course of our patient was quite different from previous case reports of paediatric IMNM. Rouster-Steven KA et al. reported 3 African American girls with anti-SRP who developed progressive weakness after systemic infection. Anti-SRP is a muscle specific autoantibody associated with severe polymyositis with interstitial lung disease, esophageal dysfunction, cardiac disease and/or Raynaud phenomenon. Clinical course was progressive despite multiple immunotherapies and resulted in a wheelchair bound condition in two patients and mild physical limitations in the third patient [7]. Mohassel P et al. described a boy with anti-HMGCR positive necrotizing myopathy who presented with chronic progressive muscle weakness over several years mimicking muscular dystrophy. The patient also developed linear scleroderma at the right lower extremity a few months before onset of muscle weakness. He was treated with IVIG monotherapy for 3 months with complete recovery [8].

Tansley SL et al. reported 4 of 381 patients (1%) of anti-HMGCR autoantibodies from a large cohort of children with idiopathic inflammatory myopathies. Four statin-naïve patients presented with slow-onset severe muscle disease and minimal cutaneous disease. All patients showed poor response to standard treatment and received biologic therapies [9]. Liang WC et al. screened anti-HMGCR in 62 children with inflammatory myopathy. Nine patients (15%) were positive. Most of the patients presented with chronic progressive weakness akin to muscular dystrophy. Early treatment of immunotherapy is deemed important for these patients [10]. Kishi et al. also reported 2 patients with anti-HMGCR IMNM, who had severe muscle weakness and only partially responded to multiple immunosuppressive medications. There was a strong association with HLA-DRB1*07:01 in anti-HMGCR-positive children in this study. In contrast, HLA-DRB1*11:01 is known to increase the risk of anti-HMGCR myopathy in adult patients [11]. A 2020 study by Verardo et al. reported on a 7-year-old boy with slow progression of muscle weakness and skin rashes who finally was diagnosed with anti-HMGCR IMNM [12]. This is the first reported case of cardiac involvement in paediatric patients with this specific autoantibody-related IMNM. From our review, all previous studies described a slower disease progression compared to our case report. The absence of skin involvement was another unique feature in our report.

We hypothesize that dengue infection may trigger host immune response and can facilitate antibody production through an unknown mechanism. This pathomechanism was previously reported in a study by Shimizu et al. describing one patient with anti-HMGCR myopathy after acute EBV infection [13]. This study also supported evidence from a previous observation by Li HM et al. in 2018 that found dengue patients were associated with having a risk of different autoimmune diseases [14].

## Conclusions

Our report presents a child with clinical manifestations of IMNM which can be categorized as acute onset of motor weakness following dengue infection. Two key points supported a diagnosis of IMNM in this patient including clinical improvement after immunosuppressive medication and the absence of typical rashes found in juvenile dermatomyositis. Findings from this report provide support for expanding the IMNM spectrum to include these types of cases. Study of additional cases is necessary to understand the pathomechanistic relationship between dengue infection and anti-HMGCR myopathy. This case review also underscores the importance of testing paediatric patients suspected of having IMNM for anti-HMGCR antibodies to help define the disease subgroup and improve understanding of the clinical course. This may lead to more timely treatment interventions with effective immunotherapies that can prevent severe disease progression.

*Myositis profile: KU, PM-Scl100, PM-Scl75, Jo-1, PL-7, PL-12, EJ, OJ, Ro-52, MI-2 Alpha, MI-2 Beta, TIF1 Gamma, MDA5, NXP2, SAE1.

## Data Availability

Not applicable.

## Abbreviations

Anti-HMGCR: Anti-3-hydroxy-3-methylglutaryl-CoA reductase
Anti SRP: Anti-signal recognition particle antibody
BSA: Body surface area
CK: Creatine kinase
EBV: Epstein-Barr virus
IMNM: Immune-mediated necrotizing
IVIG: Intra-venous immune globulin
MRI: Magnetic resonance imaging
SI: Signal intensity
T2WI: T2-weighted image
